# Tandem Repeat Insertion in African Swine Fever Virus, Russia, 2012

**DOI:** 10.3201/eid2104.141792

**Published:** 2015-04

**Authors:** Katja V. Goller, Alexander S. Malogolovkin, Sergey Katorkin, Denis Kolbasov, Ilya Titov, Dirk Höper, Martin Beer, Günther M. Keil, Raquel Portugal, Sandra Blome

**Affiliations:** Friedrich-Loeffler-Institut, Greifswald, Insel Riems, Germany (K.V. Goller, D. Höper, M. Beer, G.M. Keil, R. Portugal, S. Blome);; National Research Institute for Veterinary Virology and Microbiology, Pokrov, Russia (A.S. Malogolovkin, S. Katorkin, D. Kolbasov, I. Titov)

**Keywords:** African swine fever virus, viruses, African swine fever, intergenic region, tandem repeat sequence insertion, sequence analysis, Russia

**To the Editor:** The recent introduction of African swine fever virus (ASFV) into the European Union (http://www.oie.int/animal-health-in-the-world/the-world-animal-health-information-system/data-after-2004-wahid/) has caused serious concern in pig industries in Europe and their trade partners. African swine fever is one of the most feared infections that can affect pig industries because no vaccine is available and the socioeconomic effect of an outbreak would be serious (*1*). Therefore, early detection and coordinated countermeasures are urgently needed. For these countermeasures, information on disease dynamics and evolution is mandatory. In this respect, molecular epidemiology can be used to trace virus spread and transmission pattern.

Because it is a DNA virus, ASFV evolves rather slowly, and the use of routine genome fragments (variable region of the B646L gene and parts of the E183L gene) for partial sequencing has so far shown 100% identity among strains found in Russia (*2*) and the neighboring countries (*3*). Thus, the resolution power of these approaches is too low for in-detail analyses, which depend on information regarding larger genome fragments or whole genomes.

In 2014, an insertion of a tandem repeat sequence (TRS) in the intergenic region between the I73R and the I329L protein genes was found in ASFV strains from Poland and Lithuania (*3*). This TRS insertion was also found in ASFV strains from Ukraine in July 2012 and from Belarus in June 2013, but not in strains from Russia, Georgia, or Azerbaijan. Gallardo et al. (*3*) concluded that ASFV strains in Lithuania and Poland most likely originated from Belarus. However, these authors indicated that for a full understanding of evolution and spread, additional sequence analyses would be needed, especially from regions of Russia bordering Belarus and Ukraine. We report information for 3 additional sequences from ASFV strains from Russia that were analyzed for the previously-mentioned TRS insertion on the basis of full-genome sequences.

These ASFV strains originated from domestic pigs from the Tulskaya oblast (Tula06/2012), the Tverskaya oblast Kashinskiy district (Kashinskiy 09/2012), and the Tverskaya oblast (Tver06/2012) in 2012. Genome sequences were obtained by using a primer-walking method that was adapted from Portugal et al. (*4*). Resulting PCR products were subjected to next-generation sequencing by using the MiSeq platform (Illumina, San Diego, CA, USA). Raw sequence data were analyzed and assembled by using Genome Sequencer software version 2.6 (Roche, Mannheim, Germany). Additional sequences of the intergenic region of 17 virus isolates from domestic pigs and wild boar from Russia were obtained by using conventional PCR, and amplicons were directly sequenced by using a 3130x1 Genetic Analyzer (Applied Biosystems, Foster City, CA, USA) according to the manufacturer’s recommendations.

Chromatograms were manually edited and assembled by using CAP3 (http://pbil.univ-lyon1.fr/cap3.php). All nucleotide sequences of ASFV isolates obtained in this study were deposited in GenBank under accession nos. KP137625–KP137644. In the alignment, other published sequences available in GenBank from Poland, Lithuania, Belarus, Ukraine, Armenia, Azerbaijan, Russia, and Georgia were included (Technical Appendix). Sequence alignment was performed by using the ClustalW algorithm (http://www.clustal.org) as implemented in Geneious version 7.1.7 (Biomatters Ltd., Auckland, New Zealand).

Spatial and temporal patterns were evaluated by using a map generated with the ArcMap package implemented in ArcGIS software 10.1 (ESRI CIS Ltd., Moscow, Russia). This map shows geographic locations of virus isolates from 2011 onwards (Figure). The alignment, as well as the geographic distribution of the available isolates, clearly shows that the TRS insertion was present in 2012, especially in the Russian Tulskaya oblast (Figure). The TRS insertion predominates in subsequent isolates, although isolates without the TRS insertion are still present. Furthermore, this TRS insertion was also present in Ukraine in 2012 but could not be found in any isolates obtained in the Tverskaya Oblast in 2011 and 2012 (Figure).

**Figure:**
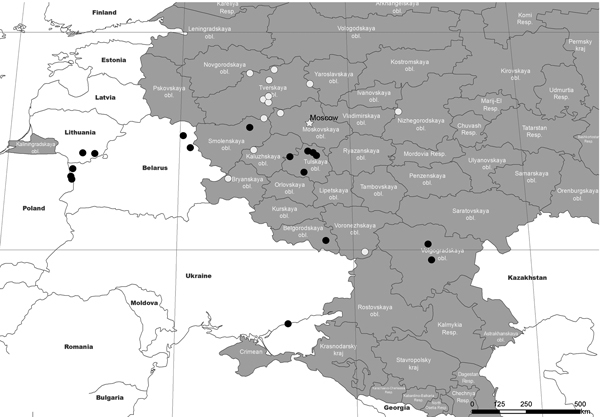
Locations where isolates of African swine fever virus were obtained in Russia during or after 2011. Black circles indicate isolates with tandem repeat insertions, and white circles indicate isolates without tandem repeat insertions. obl., oblast; Resp., respublika.

In conclusion, these findings confirm the suitability of the described TRS for a higher resolution of ASFV molecular epidemiology. However, this TRS insertion was already present in ASFV strains from Russia and is not restricted only to strains from central Europe. Thus, it can be hypothesized that viruses introduced into the European Union originated in Russia, emerged in 2012 or even earlier, and were transmitted through Belarus and Ukraine.

**Technical Appendix.** Partial nucleotide alignment of African swine fever virus intergenic regions between I73R and I329L, Russia.
