# Supplementary material for: Tandem Repeat Insertion in African Swine Fever Virus, Russia, 2012
Source: Emerg Infect Dis. 2015 Apr;21(4):731–2. doi: 10.3201/eid2104.141792 (PMC4378484; doi:10.3201/eid2104.141792)
Supplement: Supplementary file 1 — Technical Appendix. Partial nucleotide alignment of African swine fever virus intergenic regions between I73R and I329L, Russia. [file 14-1792-Techapp-s1.pdf]

Article DOI: http://dx.doi.org/10.3201/eid2104.141792

Tandem Repeat Insertion in African Swine Fever Virus, Russia, 2012

Technical Appendix. Partial nucleotide alignment of African swine fever virus intergenic regions between I73R and I329L, Russia

```
                                       10        20        30        40        50        60        70        80        90       100
                               ....|....|....|....|....|....|....|....|....|....|....|....|....|....|....|....|....|....|....|....|
1.Georgia_2007/1_FR682468      AATAACACAGTTAAGCAATAAATAACAAGTATATAGGAATATATA----------GGAATATATAGAAATATATAGAAATAGCTAAGCTTAATACTAATT
2.Abk07_KJ620029               AATAACACAGTTAAGCAATAAATAACAAGTATATAGGAATATATA----------GGAATATATAGAAATATATAGAAATAGCTAAGCTTAATACTAATT
3.Arm07_KJ620028               AATAACACAGTTAAGCAATAAATAACAAGTATATAGGAATATATA----------GGAATATATAGAAATATATAGAAATAGCTAAGCTTAATACTAATT
4.Che07_KJ620030               AATAACACAGTTAAGCAATAAATAACAAGTATATAGGAATATATA----------GGAATATATAGAAATATATAGAAATAGCTAAGCTTAATACTAATT
5.Az08D_KJ620035               AATAACACAGTTAAGCAATAAATAACAAGTATATAGGAATATATA----------GGAATATATAGAAATATATAGAAATAGCTAAGCTTAATACTAATT
6.Az08B_KJ620036               AATAACACAGTTAAGCAATAAATAACAAGTATATAGGAATATATA----------GGAATATATAGAAATATATAGAAATAGCTAAGCTTAATACTAATT
7.Ing08_KJ620031               AATAACACAGTTAAGCAATAAATAACAAGTATATAGGAATATATA----------GGAATATATAGAAATATATAGAAATAGCTAAGCTTAATACTAATT
8.Oren08_KJ620032              AATAACACAGTTAAGCAATAAATAACAAGTATATAGGAATATATA----------GGAATATATAGAAATATATAGAAATAGCTAAGCTTAATACTAATT
9.NO08/Av_KJ620033             AATAACACAGTTAAGCAATAAATAACAAGTATATAGGAATATATA----------GGAATATATAGAAATATATAGAAATAGCTAAGCTTAATACTAATT
10.NO08/Ap_KJ620034            AATAACACAGTTAAGCAATAAATAACAAGTATATAGGAATATATA----------GGAATATATAGAAATATATAGAAATAGCTAAGCTTAATACTAATT
11.Dagestan09_KJ620048         AATAACACAGTTAAGCAATAAATAACAAGTATATAGGAATATATA----------GGAATATATAGAAATATATAGAAATAGCTAAGCTTAATACTAATT
12.StPet09_KJ620050            AATAACACAGTTAAGCAATAAATAACAAGTATATAGGAATATATA----------GGAATATATAGAAATATATAGAAATAGCTAAGCTTAATACTAATT
13.Kalmykia09_KJ620049         AATAACACAGTTAAGCAATAAATAACAAGTATATAGGAATATATA----------GGAATATATAGAAATATATAGAAATAGCTAAGCTTAATACTAATT
14.Rostov09_KJ620051           AATAACACAGTTAAGCAATAAATAACAAGTATATAGGAATATATA----------GGAATATATAGAAATATATAGAAATAGCTAAGCTTAATACTAATT
15.Tver0511/Torjo_KJ620038     AATAACACAGTTAAGCAATAAATAACAAGTATATAGGAATATATA----------GGAATATATAGAAATATATAGAAATAGCTAAGCTTAATACTAATT
16.Tver0312/Torjo_KJ620041     AATAACACAGTTAAGCAATAAATAACAAGTATATAGGAATATATA----------GGAATATATAGAAATATATAGAAATAGCTAAGCTTAATACTAATT
17.Tver0312/Novo_KJ620042      AATAACACAGTTAAGCAATAAATAACAAGTATATAGGAATATATA----------GGAATATATAGAAATATATAGAAATAGCTAAGCTTAATACTAATT
18.Tver0712/Les_KJ620040       AATAACACAGTTAAGCAATAAATAACAAGTATATAGGAATATATA----------GGAATATATAGAAATATATAGAAATAGCTAAGCTTAATACTAATT
19.Tver0812/Bolo_KJ620039      AATAACACAGTTAAGCAATAAATAACAAGTATATAGGAATATATA----------GGAATATATAGAAATATATAGAAATAGCTAAGCTTAATACTAATT
20.Tver06/2012_KP137626        AATAACACAGTTAAGCAATAAATAACAAGTATATAGGAATATATA----------GGAATATATAGAAATATATAGAAATAGCTAAGCTTAATACTAATT
21.Kashinskiy09/2012_KP137627  AATAACACAGTTAAGCAATAAATAACAAGTATATAGGAATATATA----------GGAATATATAGAAATATATAGAAATAGCTAAGCTTAATACTAATT
22.Tula06/2012_KP137625        AATAACACAGTTAAGCAATAAATAACAAGTATATAGGAATATATAGGAATATATAGGAATATATAGAAATATATAGAAATAGCTAAGCTTAATACTAATT
23.Ukr12/Zapo_KJ620037         AATAACACAGTTAAGCAATAAATAACAAGTATATAGGAATATATAGGAATATATAGGAATATATAGAAATATATAGAAATAGCTAAGCTTAATACTAATT
24.Tula_06/13_KP137641         AATAACACAGTTAAGCAATAAATAACAAGTATATAGGAATATATAGGAATATATAGGAATATATAGAAATATATAGAAATAGCTAAGCTTAATACTAATT
25.Smolensk_06/13_KP137632     AATAACACAGTTAAGCAATAAATAACAAGTATATAGGAATATATA----------GGAATATATAGAAATATATAGAAATAGCTAAGCTTAATACTAATT
26.Moscow_07/13_KP137629       AATAACACAGTTAAGCAATAAATAACAAGTATATAGGAATATATA----------GGAATATATAGAAATATATAGAAATAGCTAAGCTTAATACTAATT
27.Voroneg_07/13_KP137635      AATAACACAGTTAAGCAATAAATAACAAGTATATAGGAATATATAGGAATATATAGGAATATATAGAAATATATAGAAATAGCTAAGCTTAATACTAATT
28.N.Novgorod_07/13_KP137628   AATAACACAGTTAAGCAATAAATAACAAGTATATAGGAATATATA----------GGAATATATAGAAATATATAGAAATAGCTAAGCTTAATACTAATT
29.Tula_08/13_KP137633         AATAACACAGTTAAGCAATAAATAACAAGTATATAGGAATATATAGGAATATATAGGAATATATAGAAATATATAGAAATAGCTAAGCTTAATACTAATT
30.Smolensk_09/13_KP137634     AATAACACAGTTAAGCAATAAATAACAAGTATATAGGAATATATAGGAATATATAGGAATATATAGAAATATATAGAAATAGCTAAGCTTAATACTAATT
31.Bel13/Grodno_KJ620043       AATAACACAGTTAAGCAATAAATAACAAGTATATAGGAATATATAGGAATATATAGGAATATATAGAAATATATAGAAATAGCTAAGCTTAATACTAATT
32.Tula_01/14_KP137644         AATAACACAGTTAAGCAATAAATAACAAGTATATAGGAATATATAGGAATATATAGGAATATATAGAAATATATAGAAATAGCTAAGCTTAATACTAATT
33.Kaluga_01/14_KP137639       AATAACACAGTTAAGCAATAAATAACAAGTATATAGGAATATATAGGAATATATAGGAATATATAGAAATATATAGAAATAGCTAAGCTTAATACTAATT
34.Volgograd_01/14_KP137637    AATAACACAGTTAAGCAATAAATAACAAGTATATAGGAATATATAGGAATATATAGGAATATATAGAAATATATAGAAATAGCTAAGCTTAATACTAATT
35.Tula_02/14_KP137640         AATAACACAGTTAAGCAATAAATAACAAGTATATAGGAATATATAGGAATATATAGGAATATATAGAAATATATAGAAATAGCTAAGCTTAATACTAATT
36.Volgograd_02/14_KP137642    AATAACACAGTTAAGCAATAAATAACAAGTATATAGGAATATATAGGAATATATAGGAATATATAGAAATATATAGAAATAGCTAAGCTTAATACTAATT
37.Bryansk_02/14_KP137638      AATAACACAGTTAAGCAATAAATAACAAGTATATAGGAATATATAGGAATATATAGGAATATATAGAAATATATAGAAATAGCTAAGCTTAATACTAATT
38.Bryansk_05/14_KP137630      AATAACACAGTTAAGCAATAAATAACAAGTATATAGGAATATATA----------GGAATATATAGAAATATATAGAAATAGCTAAGCTTAATACTAATT
39.Smolensk_05/14_KP137631     AATAACACAGTTAAGCAATAAATAACAAGTATATAGGAATATATA----------GGAATATATAGAAATATATAGAAATAGCTAAGCTTAATACTAATT
40.Belgorod_06/14_KP137636     AATAACACAGTTAAGCAATAAATAACAAGTATATAGGAATATATAGGAATATATAGGAATATATAGAAATATATAGAAATAGCTAAGCTTAATACTAATT
41.Kaluga_08/14_KP137643       AATAACACAGTTAAGCAATAAATAACAAGTATATAGGAATATATAGGAATATATAGGAATATATAGAAATATATAGAAATAGCTAAGCTTAATACTAATT
42.Lt14/1490_KJ620044          AATAACACAGTTAAGCAATAAATAACAAGTATATAGGAATATATAGGAATATATAGGAATATATAGAAATATATAGAAATAGCTAAGCTTAATACTAATT
43.Lt14/1482_KJ620045          AATAACACAGTTAAGCAATAAATAACAAGTATATAGGAATATATAGGAATATATAGGAATATATAGAAATATATAGAAATAGCTAAGCTTAATACTAATT
44.Pol14/Krus_KJ620047         AATAACACAGTTAAGCAATAAATAACAAGTATATAGGAATATATAGGAATATATAGGAATATATAGAAATATATAGAAATAGCTAAGCTTAATACTAATT
45.Pol14/Sz_KJ620046           AATAACACAGTTAAGCAATAAATAACAAGTATATAGGAATATATAGGAATATATAGGAATATATAGAAATATATAGAAATAGCTAAGCTTAATACTAATT
```

Technical Appendix Figure. Partial nucleotide alignment of African swine fever virus intergenic regions between I73R and I329L, Russia. -, regions without insertions. Blue indicates isolates without tandem repeat insertions, and red indicates isolates with tandem repeat insertions. Gray shading indicates isolates sequenced in this study. GenBank accession numbers are shown. Sequences in the alignment were from isolates from Poland (Pol14/Sz, Pol14/Krus), Lithuania (Lt14/1490, Lt14/1482), Belarus (Bel13/Grodno), Ukraine (Ukr12/Zap), Armenia (Arm07), Azerbaijan (Az08D, Az08B), Georgia (Georgia_2007/1, Abk07) and Russia (Che07, Ing08, Oren08, NO08/Av, NO08/Ap, Dagestan09, StPet09, Kalmykia09, Rostov09; Tver0511/Torjo, Tver0312/Novo, Tver0312/Torjo, Tver0712/Les, Tver0812/Bolo, Tver06/2012, Kashinskiy09/2012, Tula06/2012, Tula_06/13, Smolensk_06/13, Moscow_07/13, Voroneg_07/13, N.Novgorod_07/13, Tula_08/13, Smolensk_09/13, Tula_01/14, Kaluga_01/14, Volgograd_01/14, Tula_02/14, Volgograd_02/14, Bryansk_02/14, Bryansk_05/14, Smolensk_05/14, Belgorod_06/14, Kaluga_08/14).
